# *ACEF* performed better than other risk scores in non-ST-elevation acute coronary syndrome during long term follow-up

**DOI:** 10.1186/s12872-020-01841-2

**Published:** 2021-02-03

**Authors:** Ivica Kristić, Nikola Crnčević, Frane Runjić, Vesna Čapkun, Ozren Polašek, Andrija Matetic, Mislav Vrsalovic

**Affiliations:** 1 Department of Cardiology, University Hospital of Split, Split, Croatia; 2 University Department of Health Studies, University of Split, Split, Croatia; 3 Department of Public Health, School of Medicine, University of Split, Split, Croatia; 4 Department of Cardiology, Sestre Milosrdnice University Hospital Center, Vinogradska cesta 29, 10000 Zagreb, Croatia; 5 University of Zagreb School of Medicine, Zagreb, Croatia

**Keywords:** Non-ST-elevation acute coronary syndrome, Risk scores, All-treatment strategies

## Abstract

**Background:**

Risk stratification of patients with non-ST-elevation acute coronary syndrome (NSTE-ACS) is an important clinical method, but long-term studies on patients subjected to all-treatment strategies are lacking. Therefore, the aim was to compare several established risk scores in the all-treatment NSTE-ACS cohort during long-term follow-up.

**Methods:**

Consecutive patients (n = 276) with NSTE-ACS undergoing coronary angiography were recruited between September 2012 and May 2015. Six risk scores for all patients were calculated, namely *GRACE 2.0, ACEF, SYNTAX, Clinical SYNTAX, SYNTAX II PCI and SYNTAX II CABG*. The primary end-point was Major Adverse Cardiovascular Events (MACE) which was a composite of cardiac death, nonfatal myocardial infarction, ischemic stroke or urgent coronary revascularization.

**Results:**

During a median follow-up of 33 months, 64 MACE outcomes were recorded (23.2%). There was no difference between risk score categories, except in the highest risk group of *ACEF* and *SYNTAX II PCI* scores which exhibited significantly more MACE (51.6%, N = 33 and 45.3%, N = 29, *P* = 0.024, respectively). In the multivariate Cox regression analysis of individual variables, only age and atrial fibrillation were significant predictors for MACE (HR 1.03, 95% CI 1.00–1.05, *P* = 0.023 and HR 2.02, 95% CI 1.04–3.89, *P* = 0.037, respectively). Furthermore, multivariate analysis of the risk scores showed significant prediction of MACE only with *ACEF* score (HR 2.16, 95% CI 1.36–3.44, *P* = 0.001). The overall performance of *GRACE*, *SYNTAX*, *Clinical SYNTAX* and *SYNTAX II CABG* was poor with AUC values of 0.596, 0.507, 0.530 and 0.582, respectively, while *ACEF* and *SYNTAX II PCI* showed the best absolute AUC values for MACE (0.630 and 0.626, respectively).

**Conclusions:**

*ACEF* risk score showed better discrimination than other risk scores in NSTE-ACS patients undergoing all-treatment strategies over long-term follow-up and it could represent a fast and user-friendly tool to stratify NSTE-ACS patients.

## Background

Non-ST-elevation acute coronary syndrome (NSTE-ACS) is one of the leading causes of cardiovascular morbidity and mortality, with a rising prevalence in the last decades [1, 2]. It is comprised out of two closely interconnected clinical entities, namely non-ST-segment elevation myocardial infarction (NSTEMI) and unstable angina (UA) [3, 4]. Improved preventive measures and higher sensitivity of diagnostic methods have led to a decreased incidence of ST-segment elevation myocardial infarction (STEMI) with a relative increase of NSTE-ACS events in the total cohort of acute coronary syndrome (ACS) patients [1].

However, ACS represents a heterogeneous syndrome with significantly different outcomes among its subgroups and subpopulations [5, 6]. While short-term outcomes are worse in STEMI patients due to a higher rate of in-hospital mortality, NSTE-ACS patients generally exhibit worse long-term adverse outcomes [6]. Furthermore, subgroups of NSTE-ACS (NSTEMI and UA) also differ in long-term prognosis [5], and the appropriate management of these patients still represents a subject of debate [7]. Therefore, the risk stratification of these patients helps to establish the most appropriate therapeutic strategy with short-term and long-term prognostic implications [3, 4]. Recent studies and guidelines advocate that specific high-risk subgroups may benefit from an aggressive therapeutic approach in NSTE-ACS [3, 4].

According to the current guidelines, quantitative risk assessment using a clinical risk score *Global Registry for Acute Coronary Events* (*GRACE*) may be considered for the prognostic estimation of NSTE-ACS patients [4, 8, 9], while other risk scores like *Age, Creatinine, Ejection Fraction* (*ACEF*), *The Synergy Between Percutaneous Coronary Intervention with TAXUS and Cardiac Surgery* (*SYNTAX*), *Clinical SYNTAX* and *SYNTAX II* score have been previously investigated in similar clinical settings as well [10–14]. However, comprehensive studies comparing the performance of the aforementioned risk scores in NSTE-ACS patients with long-term follow-up are lacking. Therefore, the aim was to compare the long-term discrimination and calibration of several clinical and angiographic risk scores, namely *GRACE 2.0*, *ACEF*, *SYNTAX*, *Clinical SYNTAX*, *SYNTAX II* for percutaneous coronary intervention (PCI) and *SYNTAX II* for coronary artery bypass grafting (CABG), in the NSTE-ACS cohort undergoing all treatment strategies including PCI, CABG or conservative management.

## Methods

### Ethical and institutional considerations

All the proceedings and clinical research were performed in accordance with the ethical standards and amendments of the Declaration of Helsinki. The study protocol was approved by the Ethical Committee of the University Hospital of Split, Croatia (No. 2181-147-01/06). All the participants included in the study provided formal written informed consent for coronary angiography and informed verbal consent for the use of relevant medical data, which is in accordance with the Approval of the Ethical committee of the University Hospital of Split and the Declaration of Helsinki. All participants were informed about the goal and course of this study.

### Study design

This was a single-centre observational prospective study. All patients with NSTE-ACS undergoing a coronary angiography at the University Hospital of Split between September 2012 and May 2015 were considered eligible. Patients with active malignant disease and a history of CABG were excluded (Additional file 1: Fig. S1). All patients, including those receiving a conservative treatment, underwent coronary angiography. Baseline characteristics were obtained from electronic health records. The diagnosis of NSTE-ACS (UA and NSTEMI) was established according to the competent international guidelines [3, 4]. The reasons for loss of follow-up were inability to contact a patient, or patient refusal of further follow-up. All patients were followed up through scheduled clinical visits or telephone interviews firstly 3 months after the index event and thereafter at a 12-month interval, with a final contact in May 2017.

### Laboratory analysis

Blood samples were collected from all participants included in the study. These were used to measure cardiac troponin I, with a threshold for positivity of 0.033 ng/ml and other usual biochemical parameters. All laboratory analyses were done in the same biochemical laboratory and measured by standard laboratory methods. Glomerular filtration rate (eGFR) was estimated using a Cockcroft–Gault formula.

### Treatment strategies

Coronary angiography was primarily performed over radial access. After a diagnostic coronary angiogram was performed, patients were treated with PCI, CABG or conservatively based on the heart team decision and patient preferences. All patients were treated with a tailored treatment plan, which included dual antiplatelet therapy, in line with the current guidelines [4].

### Outcomes

A key primary endpoint included Major Adverse Cardiac Event (MACE), which was a composite of cardiac death, nonfatal myocardial infarction (MI), ischemic stroke or urgent coronary revascularization. All deaths were considered to be of cardiac origin unless sufficient evidence indicated a non-cardiac cause of death. Nonfatal MI was defined as a recurrent MI with or without ST-elevation or UA. UA was included among MI events only if there was an angiographic confirmation of an unstable lesion and a subsequent revascularization. Urgent coronary revascularization was defined as urgent intervention, percutaneous or surgical, due to highly symptomatic stable angina. All outcomes were evaluated by a team of experienced cardiologists (I.K., F.R. and M.V.).

### Clinical and risk assessment

Individual risk was determined for all participants included in the study. Risk assessment was conducted by six established risk scores: *GRACE 2.0*, *ACEF*, *SYNTAX*, *Clinical SYNTAX*, *SYNTAX II for PCI* and *SYNTAX II for CABG*. Necessary information was extracted from patient medical records, electrocardiograms, laboratory analysis, and angiographic data during initial hospitalization. Anthropometric data were collected according to the standard methods.

The *GRACE* score was calculated using an online calculator version 2.0 [8]. The three-variable *ACEF* model was calculated according to the following formula: age (years)/left ventricular ejection fraction (percentage) + 1 (if serum creatinine > 176 µmol/L [> 2 mg/dL]) [15]. The *SYNTAX* score was computed from the baseline coronary angiogram, as previously described, by two experienced interventional cardiologists (I.K. and N.C.). In case of disagreement, a third cardiologist re-evaluated coronary angiogram (M.V.) [11]. The *Clinical SYNTAX* score was calculated by multiplying the values of *ACEF* and the *SYNTAX* score [13]. The *SYNTAX II* score was calculated using the online calculator, as previously described [16]. The overview of the used risk scores is presented in the Additional file 2: Table S1 and S2.

Patients were stratified in different groups according to the risk score values. The following thresholds were used—*GRACE* score: < 88.0, 8.0–118.0, > 119.0; *ACEF* score (tertiles): < 1.0, 1.0–1.24, > 1.24; *SYNTAX* score: ≤ 22, 23–32, ≥ 33; *Clinical SYNTAX* score (tertiles): < 10.42, 10.42–23.9, > 23.9; *SYNTAX score II for PCI* (tertiles): < 22.7, 22.7–31.6, > 31.6; *SYNTAX score II for CABG* (tertiles): < 20.6, 20.6–30.8, > 30.8. Cut-off values for *GRACE* score and *SYNTAX* score were set according to previously established criteria for 6-month post-discharge mortality and complexity of coronary disease, respectively [11, 17]. In addition, a novel model has been constructed that encompassed three independent predictor variables from the multivariate analysis: *ACEF*, female gender and atrial fibrillation, and was compared with other risk scores.

### Statistical analysis

Statistical analysis was conducted according to standard statistical methods. The normality of data distribution was assessed by the Kolmogorov–Smirnov test. Continuous data were presented as mean ± standard deviation (SD) or as median (interquartile range, IQR). Student's T-test or Mann–Whitney U test were used for continuous data analysis according to parametric or non-parametric distribution, respectively. Categorical variables were expressed as numbers and percentages and analysed using the Chi-squared test. The accuracy of each variable in predicting MACE was tested using receiver operating characteristic (ROC), with a calculation of area under the curve (AUC). The reported *P* values represent the significance relative to the non-informative ROC curve (AUC = 0.5) and were tested using a SPSS algorithm based on methodology of *Hanley* and *McNeil* [18]. The cumulative incidence of MACE was estimated using the Kaplan–Meier approach, and significance was assessed using the Mantel-Cox log-rank test. Cox logistic regression analysis was performed to determine the predictors of MACE in the univariate and the multivariate model. A separate multivariate model of the individual variables and risk scores have been conducted to avoid multicollinearity. A multivariate analysis of individual variables contained all variables with a *P* < 0.1 at univariate analysis, while the multivariate analysis of risk scores retained only *ACEF*, *GRACE* and *SYNTAX II PCI* score. The multivariate analysis tested the 10-unit change for *GRACE* and *SYNTAX II PCI* scores. The *SYNTAX*, *Clinical SYNTAX* and *SYNTAX II CABG* scores have been excluded from the multivariate analysis to avoid multicollinearity. A stepwise forward algorithm (with a removal criterion set to *P* < 0.1) was used in the multivariate analysis. The results of the risk analyses are provided as hazard ratio (HR) and 95% confidence intervals (95% CI) which corresponds to a 1-unit increase/decrease of each score on a continuous scale. Furthermore, additional HR for all scores (except *ACEF*) have been presented from the univariate analysis which correspond to a 10-unit increase/decrease of each score. Calibration, a measure of the agreement between observed and predicted outcomes, was assessed by the Hosmer–Lemeshow goodness-of-fit test. A two-sided *P*-value of < 0.05 was considered significant. Statistical data analysis was carried out using a Statistical Package for the Social Sciences (SPSS) software (IBM Corp, NY, USA; version 20).

## Results

A total of 300 patients were initially enrolled in the study protocol, of which 276 completed follow-up. The study population was mostly comprised of older adult male patients (64.3 ± 11.4 years and 74.3%, N = 205, respectively). The femoral access was used in only 7.4% patients all others were treated via radial approach. The median follow-up period was 35 months (IQR 26–42 months). Of the 276 patients who completed follow-up, MACE occurred in 64 patients (23.2%), including 16 cardiac deaths (5.8%), 10 MI (3.6%), 4 ischemic strokes (1.4%), and 34 urgent coronary revascularizations (12.3%). Patients who developed MACE were significantly older and showed a higher prevalence of female patients and atrial fibrillation, but exhibited lower values of BMI, haemoglobin, eGFR and left ventricular ejection fraction (*P* < 0.05). There was no statistically significant difference in other baseline and laboratory parameters (Table 1).

**Table 1:** Comparison of baseline anthropometric, laboratory and echocardiographic parameters of study participants

Variables	MACE		Total (n = 276)	*P* value
	No (n = 212)	Yes (n = 64)		
Age (years)	63.2 ± 10.9	68.0 ± 11.1	64.3 ± 11.4	0.002*
Female gender	47 (22.2)	24 (37.5)	71 (25.7)	0.014^†^
BMI (kg/m^2^)	27.8 (25.8–30.9)	26.6 (24.4–29.8)	27.8 (25.4–30.7)	0.026^‡^
NSTE-ACS subtype				0.733^†^
NSTEMI	175 (82.5)	54 (84.4)	229 (83.0)	
UA	37 (17.5)	10 (15.6)	47 (17.0)	
Arterial hypertension	131 (61.8)	46 (71.9)	177 (64.1)	0.140^†^
Diabetes mellitus	60 (28.3)	23 (35.9)	83 (30.1)	0.110^†^
Hypercholesterolemia	106 (50.0)	32 (50.0)	138 (50.0)	0.999^†^
Family history of CVD	76 (35.8)	25 (39.1)	101 (36.6)	0.640^†^
Active smoking	62 (29.2)	14 (21.9)	76 (27.5)	0.260^†^
Atrial fibrillation	15 (7.1)	11 (17.2)	26 (9.4)	0.015^†^
Prior CAD	8 (3.8)	4 (6.3)	12 (4.3)	0.395^†^
Prior MI	19 (9.0)	11 (17.2)	30 (10.9)	0.066^†^
Prior PCI	17 (8.0)	6 (9.4)	23 (8.3)	0.731^†^
PAD	20 (9.5)	9 (14.1)	29 (10.5)	0.432^†^
COPD	26 (12.2)	7 (10.9)	33 (12.0)	0.920^†^
Killip class > 1	20 (9.0)	8 (12.5)	28 (9.8)	0.692^†^
Hgb (g/L)	143.0 (134.0–153.0)	137.0 (123.3–149.0)	141.2 (133.0–152.0)	0.010^‡^
Glucose (mmol/L)	7.1 (6.0–9.9)	7.4 (6.1–9.6)	7.1 (6.0–9.7)	0.672^‡^
CRP (mmol/L)	5.1 (2.0–10.5)	8.6 (1.5–29.3)	5.7 (1.7–12.9)	0.128^‡^
eGFR (ml/min)	91.8 ± 27.1	81.9 ± 28.3	89.5 ± 27.7	0.017*
LVEF (%)	59.0 (55.0–65.0)	55.0 (51.3–62.0)	58.0 (55.0–65.0)	0.046^‡^
Follow-up (months)	36.1 (29.5–43.8)	32.6 (26.5–43.5)	35.1 (29.3–44.0)	0.089^‡^

Data are expressed as mean ± SD, number (percent) or median (interquartile range)

BMI—body mass index; CAD—coronary arterial disease; COPD—chronic obstructive pulmonary disease; CRP—C-reactive peptide; CVD—cardiovascular disease; eGFR—estimated glomerular filtration rate; Hgb—hemoglobin; LVEF—left ventricular ejection fraction; MACE—major adverse cardiovascular events; MI—myocardial infarction; NSTE-ACS—non-ST elevation acute coronary syndrome; NSTEMI—non ST segment elevation myocardial infarction; UA—unstable angina; PAD—peripheral arterial disease; PCI—percutaneous coronary intervention

*Student’s T test; ^†^Chi-square test; ^‡^Mann–Whitney U test

There was no statistically significant difference in treatment strategies, features of the coronary angiogram, discharge therapy and risk scores between the different subgroups, except in the values of *ACEF*, *SYNTAX II PCI* and *SYNTAX II CABG* score which were significantly higher in patients who developed MACE (*P* < 0.05) (Table 2). Furthermore, there was no significant difference in MACE occurrence among the different NSTE-ACS risk categories, except among the *ACEF* and *SYNTAX II PCI* risk categories in which MACE occurred significantly more often in the group with the highest values (51.6%, N = 33 and 45.3%, N = 29, *P* = 0.020, respectively) (Additional file 2: Table S3). Consistently, the binomial logistic regression model revealed that patients from the highest *ACEF* and *SYNTAX II PCI* group have significantly higher odds of MACE (OR 2.76, 95% CI 1.55–4.91, *P* = 0.001; and OR 1.96, 95% CI 1.10–3.48, *P* = 0.022) in comparison to lower tertiles, while there was no statistically significant difference between risk categories for other risk scores.

**Table 2:** Comparison of therapeutic and angiographic characteristics of study participants

Variables	MACE		Total (n = 276)	*p* value
	No (n = 212)	Yes (n = 64)		
Treatments				0.071^†^
Conservative	43 (20.3)	20 (31.3)	63 (22.8)	
PCI	83 (39.2)	27 (42.2)	110 (39.9)	
CABG	86 (40.6)	17 (26.6)	103 (37.3)	
Coronary angiogram				
Left main disease	27 (12.7)	5 (7.8)	32 (11.6)	0.281^†^
Single-vessel disease	82 (38.7)	23 (35.9)	105 (38.0)	0.692^†^
Two-vessel disease	39 (18.4)	17 (26.6)	56 (20.3)	0.155^†^
Three-vessel disease	79 (37.3)	20 (31.3)	99 (35.9)	0.177^†^
Discharge therapy				
Beta blockers	158 (74.5)	42 (65.6)	200 (72.5)	0.162^†^
ACE-I/ARB	132 (62.)	44 (68.8)	176 (63.8)	0.344^†^
ASA	199 (93.9)	60 (93.8)	259 (93.8)	0.973^†^
P2Y12 inhibitors	182 (85.8)	50 (78.1)	232 (84.1)	0.139^†^
Statins	196 (92.5)	56 (87.5)	252 (91.3)	0.218^†^
Risk scores				
GRACE 2.0	99.0 (80.3–116.0)	107.5 (88.5–127.3)	100.0 (82.0–120.0)	0.072*
ACEF	1.1 (0.9–1.3)	1.3 (0.9–1.4)	1.2 (0.9–1.3)	0.002^‡^
SYNTAX	14.0 (8.0–23.4)	13.3 (7.0–26.0)	14.0 (8.0–24.0)	0.869^‡^
Clinical SYNTAX	14.8 (8.6–26.9)	15.1 (8.4–31.0)	14.9 (8.6–27.7)	0.462^‡^
SYNTAX II PCI	25.5 (20.4–33.7)	29.8 (24.1–38.5)	26.8 (20.8–34.8)	0.001*
SYNTAX II CABG	24.9 (17.2–32.0)	27.0 (18.7–36.4)	25.5 (18.1–33.6)	0.038*

Data are expressed as number (percent) or median (interquartile range)

ACE-I—angiotensin converting enzyme inhibitors; ACEF—Age, Creatinine and Ejection Fraction risk score; ARB—angiotensin receptor blockers; ASA—acetylsalicylic acid; CABG—coronary artery bypass grafting; GRACE—Global Registry of Acute coronary events risk score; MACE—major adverse cardiovascular events; PCI—percutaneous coronary intervention; SYNTAX—The Synergy Between Percutaneous Coronary Intervention with TAXUS and Cardiac Surgery risk score

*Student’s T test; ^†^Chi-square test; ^‡^Mann–Whitney U test

**Fig. 1:**
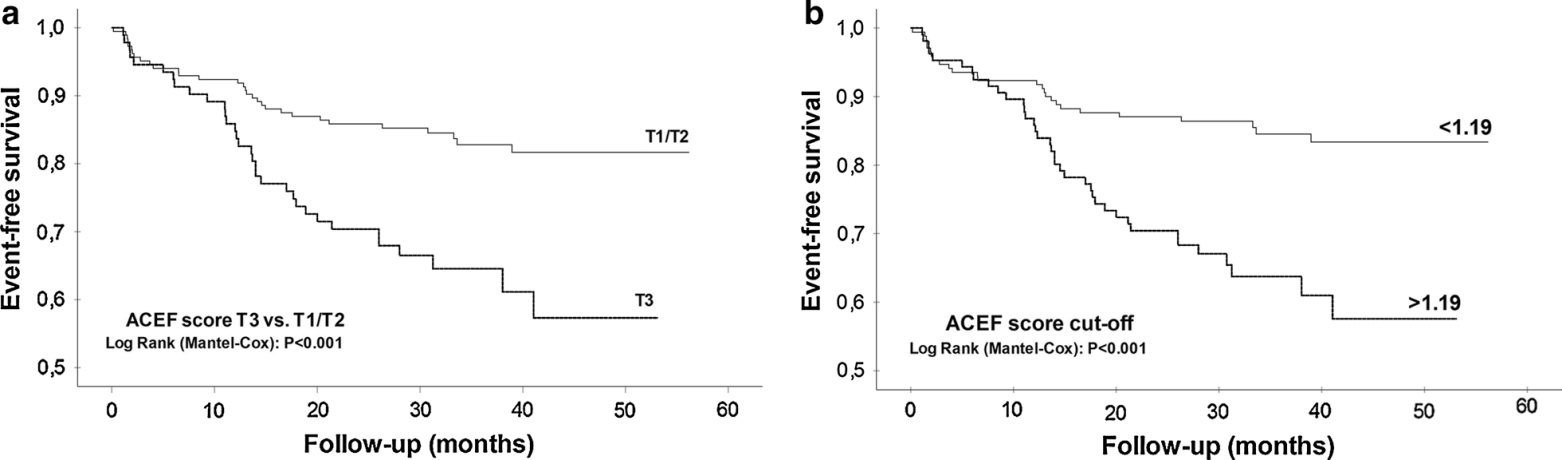
Event-free survival for MACE: **a** ACEF tertiles (T1/T2 vs. T3); **b** Optimal prognostic cut-off value*. T1—ACEF risk score first tertile (values < 1.00); T2—ACEF risk score second tertile (values 1.00–1.24); T3—ACEF risk score third tertile (values > 1.24); *determined by Youden index (< 1.19 vs. > 1.19)

Among individual variables, age, BMI, eGFR, female gender and atrial fibrillation, were significantly associated with an increased incidence of MACE in univariate analysis (*P* < 0.05), but the only significant independent predictors for MACE in the following multivariate Cox regression analysis proved to be age (HR 1.03, 95% CI 1.00–1.05, *P* = 0.023) and atrial fibrillation (HR 2.02, 95% CI 1.04–3.89, *P* = 0.037). Furthermore, in the multivariate model containing *ACEF*, *GRACE* and *SYNTAX II PCI* score, *ACEF* proved to be the only significant independent predictor for MACE (HR 2.16, 95% CI 1.36–3.44, *P* = 0.001). There was no significant association of treatment strategy with MACE (Table 3). The cumulative incidence of MACE was greater in the patients within the highest *ACEF* tertile (score > 1.24) and above the optimal *ACEF* cut-off value (score > 1.19), with approximately 10-months earlier MACE development during follow-up (38.2, 95% CI 34.1–42.3 vs. 48.5, 95% CI 46.0–51.0 months, *P* < 0.001; and 38.4, 95% CI 34.6–42.2 vs. 49.1, 95% CI 46.5–51.6 months, *P* < 0.001, respectively) (Fig. 1).

**Table 3:** Predictors of MACE

A. Individual variables (w/o risk scores)				
Variables	Univariate Cox analysis		Multivariate Cox analysis	
	HR (95% CI)	*P* value	HR (95% CI)	*P* value
Age	1.04 (1.01–1.06)	0.004	1.03 (1.00–1.05)	0.023
BMI	0.93 (0.87–0.99)	0.030	ns^‡^	
eGFR	0.99 (0.98–1.00)	0.009	ns^‡^	
Female gender	1.86 (1.12–3.09)	0.016	1.67 (1.00–2.79)	0.051
Atrial fibrillation	2.24 (1.17–4.29)	0.015	2.02 (1.04–3.89)	0.037
Previous MI	1.75 (0.92–3.35)	0.091	ns^‡^	
Conservative treatment vs. revascularization	1.53 (0.90–2.60)	0.114	n/a^§^	

B. Risk scores				
Variables	Univariate Cox analysis		Multivariate Cox analysis	
	HR (95% CI)	*P* value	HR (95% CI)	*P* value
GRACE	1.01 (1.00–1.02)*	0.088	0.97 (0.86–1.10)^‖^	0.664
	1.09 (0.99–1.19)^†^	0.076		
ACEF	2.16 (1.36–3.44)*	0.001	2.16 (1.36–3.44)^¶^	0.001
SYNTAX	1.00 (0.98–1.03)*	0.880	n/a^§^	
	0.99 (0.79–1.25)^†^	0.948		
Clinical SYNTAX	1.01 (1.00–1.03)*	0.063	n/a^§^	
	1.14 (0.99–1.29)^†^	0.059		
SYNTAX II PCI	1.04 (1.01–1.06)*	0.001	1.22 (0.88–1.69)^‖^	0.237
	1.37 (1.11–1.79)^†^	0.004		
SYNTAX II CABG	1.02 (1.00–1.04)*	0.040	n/a^§^	
	1.22 (1.00–1.50)^†^	0.053		

ACEF—age, creatinine and ejection fraction risk score; BMI—body mass index; CABG—coronary artery bypass graft; eGFR—estimated glomerular filtration rate; GRACE—Global Registry of Acute coronary events risk score; LVEF—left ventricular ejecton fraction; MACE—major adverse cardiovascular events; MI—myocardial infarction; PCI—percutaneous coronary intervention; SYNTAX—The Synergy Between Percutaneous Coronary Intervention with TAXUS and Cardiac Surgery risk score

^*^HR value corresponds to each 1 unit increase/decrease of each score

^†^HR value corresponds to each 10 unit increase/decrease of each score

^‡^ns indicates variables which were not held in the model due to statistical insignificance (stepwise forward algorithm)

^§^n/a indicates variables which were a priori not included in the multivariate analysis due to *P* > 0.1 and/or multicollinearity

^‖^The intermediate step of the multivariate analysis (stepwise forward algorithm)

^¶^The final step of the multivariate analysis (stepwise forward algorithm)

**Fig. 2:**
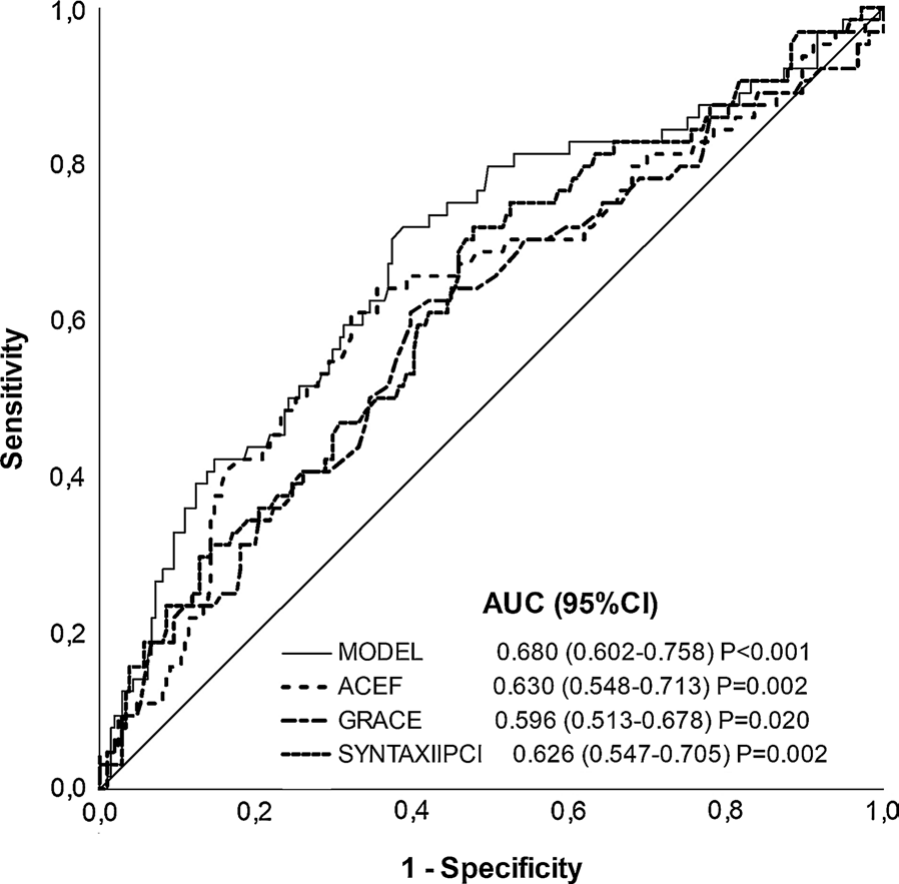
Receiver operating characteristic of predicting MACE for MODEL*, ACEF, SYNTAX II PCI and GRACE score. *Computation of ACEF, female gender and atrial fibrillation; ^†^*P* values represent the significance relatively to the non-informative ROC curve (AUC = 0.5) and were tested using a SPSS algorithm based on methodology of *Hanley* and *McNeil*; ACEF—Age, Creatinine and Ejection Fraction risk score; GRACE—Global Registry of Acute coronary events risk score; LVEF—left ventricular ejection fraction; PCI—percutaneous coronary intervention; SYNTAX—The Synergy Between Percutaneous Coronary Intervention with TAXUS and Cardiac Surgery risk score

The *ACEF* and *SYNTAX II PCI* risk score had the best discriminatory accuracy to predict MACE, with an AUC value of 0.630 (*P* = 0.002) and 0.626 (*P* = 0.002), respectively. The sensitivity and specificity were 60.9% and 67.9%, respectively for the value of the *ACEF* score of 1.19. Overall performance of *GRACE*, *SYNTAX*, *Clinical SYNTAX* and *SYNTAX II CABG* was worse with AUC values of 0.596 (*P* = 0.020), 0.507 (*P* = 0.869), 0.530 (*P* = 0.462) and 0.582 (*P* = 0.058), respectively. The best absolute AUC value of 0.680 (*P* < 0.001), which improved overall accuracy, showed the constructed model in which *ACEF*, female gender and atrial fibrillation were combined (Fig. 2). A post-hoc analysis of the computed predicted probabilities and AUC values revealed a relatively greater contribution of the female gender in the model (Additional file 2: Tables S4 and S5). Furthermore, a post-hoc ROC comparison revealed that AUC value of the constructed model does not differ statistically significantly from *ACEF* (*P* = 0.141) and *SYNTAX II PCI* (*P* = 0.123), while AUC value of *ACEF* does not differ statistically significantly from *GRACE* (*P* = 0.342) and *SYNTAX II PCI* (*P* = 0.912), but are both better in comparison to other risk scores (*P* < 0.05). The Hosmer–Lemeshow test proved adequate calibration for predicting rates of MACE: *ACEF* (χ2 15.77, *P* = 0.056), *SYNTAX II PCI* (χ2 5.01, *P* = 0.757, *GRACE* (χ2 6.63, *P* = 0.578), *SYNTAX* (χ2 5.37, *P* = 0.615), *Clinical SYNTAX* (χ2 5.82, *P* = 0.667), *SYNTAX II CABG* (χ2 12.98, *P* = 0.113) and constructed model (χ2 17,11, *P* = 0.059).

## Discussion

The risk stratification of NSTE-ACS patients represents a crucial role in everyday clinical settings. However, head-to-head comprehensive studies comparing the most utilized available risk scores in NSTE-ACS patients undergoing all-treatment strategies (PCI, CABG or conservatively), with long-term follow-up, are lacking. To our knowledge, only Stahli et al. and Palmerini et al. have questioned the role of several risk scores on 1-year outcomes in the NSTE-ACS patients managed by revascularization methods [10, 19]. This study demonstrates a comparison of *GRACE 2.0*, *ACEF*, *SYNTAX*, *Clinical SYNTAX*, *SYNTAX II PCI* and *SYNTAX II CABG* in NSTE-ACS patients undergoing all-treatment strategies, over a remarkably long-term follow-up (up to 56 months).

This study has established several key findings. First, patients who developed MACE had statistically significantly increased *ACEF* and *SYNTAX II* scores. Second, *ACEF* and *SYNTAX II PCI* proved the best discriminatory accuracy in predicting MACE, with an acceptable sensitivity and specificity ratio. Finally, the cumulative incidence of MACE was statistically significantly greater in the patients within the highest *ACEF* tertile and above the optimal *ACEF* cut-off value with approximately 10-months earlier MACE development.

*ACEF* risk score incorporates only three variables, thereby representing one of the simplest scores in terms of assessment [10, 15]. Risk assessment using an angiography-related risk scores was proposed as well, with *SYNTAX* score being the first fully-based on angiographic features [11, 12]. However, the absence of clinical variables has contributed to substantial limitations of the *SYNTAX* score [11, 13]. To overcome these limitations *Clinical SYNTAX* risk score has been developed by multiplying values of modified *ACEF* and *SYNTAX* score [13]. Further improvement was the inclusion of additional clinical and anatomic variables resulting in the *SYNTAX II* score [14]. Although these scores were developed to estimate patient prognosis and to provide optimal patient-oriented treatment, they are still largely underused [20]. Some of the main concerns are their development and validation in different clinical settings, across the entire spectrum of both stable and unstable patients, managed with different treatment strategies [21–24]. Furthermore, their prognostic strength was evaluated in different time-frames, from in-hospital outcomes to short-term and long-term follow-up [6, 19]. Finally, the complexity of risk scores aggravates everyday clinical usage indicating the importance of simple clinical scores [20].

Therefore, features of *ACEF* like simplicity of application, easily accessible components, and time-sparing properties offer potential benefits for the clinicians [15] making these results encouraging. Previous studies have reported a risk stratification role of *ACEF* in different clinical settings [25–27], but research on ACS patients have been mostly based on a heterogeneous sample of PCI-treated patients [23, 24]. The findings of good predictive value of *SYNTAX II PCI* score in this population is likewise reassuring given that it’s clinical attributes (age, creatinine clearance and left ventricular ejection fraction) are in fact the components of *ACEF* score [16].

Large *Acute Catheterization and Urgent Intervention Triage Strategy* (ACUITY) trial compared several risk scores in the prediction of 1-year clinical adverse outcomes amongst NSTE-ACS population treated with PCI. They established the best predictive accuracy for combined clinical and angiographic scores (AUC 0.60) which is followed by purely angiographic *SYNTAX* score (AUC 0.59), but only poor to modest discrimination strength of fully-clinical *ACEF* (AUC 0.52) and *GRACE* (AUC 0.52) scores [19]. Similar findings were reported in the LEADERS Trial amongst *all-comers* population undergoing PCI which showed AUC values of 0.62 and 0.58, for *Clinical SYNTAX* and *ACEF* risk scores, respectively [24]. On the contrary, the present study has shown the best discriminatory accuracy for MACE prediction with *ACEF* and *SYNTAX II PCI* scores, while *GRACE*, *SYNTAX* and *Clinical SYNTAX* had poorer overall performance. In general, this study exhibited higher absolute MACE-related AUC values for *ACEF*, similar for *GRACE*, but lower for *SYNTAX* and *Clinical SYNTAX* scores, in comparison to the ACUITY trial [19]. The poor overall performance of the *GRACE* score in this long-term study is consistent with previous studies and could be explained by its initial development for the prediction of short-term events [28, 29]. A similar study by Stahli et al. compared the predictive role of *ACEF* and *GRACE* scores on 30-day and 1-year outcomes in the total cohort of ACS patients treated with PCI or CABG. Complementary to the present study, they showed a significant independent association of the highest *ACEF* group with 1-year MACCE (HR 3.75, 95% CI 2.56–5.49) [10]. Furthermore, the data from the *Korean Acute Myocardial Infarction Registry* comprising a total ACS cohort undergoing PCI showed that the *ACEF* score was an independent predictor of 1-year mortality with a robust AUC of 0.79 [30]. However, as with a study by Stahli et al., more than half of a study sample was encompassed by STEMI patients which are known to have less benefit from risk stratification (61.3% and 53.6%, respectively) [10, 30]. Additionally, they only enrolled patients referred for coronary revascularization, with CABG rate being presumably low (~ 4%). The exclusion of pharmacologically- and small proportion of surgically-treated patients in these studies represents a possible limitation for applying these results to the general NSTE-ACS population [10, 19, 30].

Several factors could explain the findings of respectable AUC values of purely clinical risk score, while previous studies have demonstrated the predominance of combined clinical and angiographic risk scores [19, 24]. Firstly, a different study population which encompassed only NSTE-ACS patients which were treated with all management strategies could be less affected by the angiographic findings. Furthermore, longer follow-up in the present study could diminish the influence of angiographic differences from *SYNTAX*-related scores or acute setting parameters which are components of *GRACE* score (cardiac arrest on admission; Killip class; abnormal cardiac enzymes; ECG changes). It is possible that age, kidney function and cardiac capacity, even though determined by crude parameters, have similar influence on short-term and long-term outcomes, while the inclusion of other parameters have more impact on the treatment strategy and short-term prognosis. Finally, most previous similar studies have used a composite outcome consisting of all-cause mortality or included major bleeding which were not assessed by this study. This fact could also play a certain role in the discriminatory power of the risk scores. Nevertheless, these findings indicate that *ACEF* could possibly performs good enough in comparison to other complex risk scores and may serve as a fast and user-friendly tool to stratify NSTE-ACS patients.

The median follow-up of the present study was 33 months with a maximal period of up to 56 months. None of the available studies provided a similar follow-up period and insights into the role of *ACEF* in the prediction of such extended long-term outcomes. While the aforementioned studies have mostly provided insights for the 1-year outcomes, only Chichareon et al. have reported longer follow-up in all-comers in the GLOBAL LEADERS study [23].

Consistent with previous studies, the present analysis has revealed that female NSTE-ACS patients were more likely to develop adverse outcomes. Gender disparities in outcomes after MI have been reported in several studies but surpass the horizons of this paper [31, 32]. Nevertheless, a modest improvement in accuracy of *ACEF* was obtained with the addition of female gender and atrial fibrillation in the computed model in this study (AUC 0.680). These findings are not surprising since atrial fibrillation is a well-established negative prognostic factor [33].

As with the other studies, this study has several limitations. A small sample size with a relatively low incidence of end-points does not allow for additional sub-analyses, while the statistically significant low absolute values of C-statistics require careful interpretation of the true clinical significance. The composite outcome MACE included cardiac death, nonfatal MI, ischemic stroke, and urgent coronary revascularization which may impede its comparison to other studies. Furthermore, this study encompassed a relatively low-risk population of NSTE-ACS patients with a large heterogeneity in follow-up which is reflected by basal patient characteristics and aggravates its extrapolation to the total NSTE-ACS population. Similarly, the results are not applicable to patients with active malignancy or previous CABG, as they were excluded. Moreover, the study was conducted before the era of high-sensitivity cardiac troponin assays, and only NSTE-ACS patients who underwent coronary angiography were included. *SYNTAX II* scores were used in the clinical setting in which they were not validated, i.e. *SYNTAX II PCI* in patients undergoing CABG or conservative management and *SYNTAX II CABG* in patients undergoing PCI or conservative management [14]. Finally, *ACEF* categories in this study were created using original score tertiles which impede result comparisons and inter-analyses with some other studies.

In conclusion, a simple clinical risk score *ACEF* exhibited better discrimination compared to other complex risk scores, in NSTE-ACS patients undergoing all-treatment strategies over the long-term follow-up. Therefore, *ACEF* could possibly represent a fast and user-friendly tool to stratify NSTE-ACS patients. Future long-term prospective studies are necessary to strengthen this association and determine other clinical elements which might improve prognostic strength in this patient population.

## Supplementary Information

**Additional file 1. Fig. S1**: Flow diagram of the study design.

**Additional file 2. Table S1**: Overview of the first risk score validation in the setting of ACS or PCI. **Table S2**: Description of risk scores. **Table S3**: Association of risk scores with occurrence of major adverse cardiovascular events. **Table S4**: Comparison of predicted probabilities of ACEF and different computed models (derived from bivariate logistic regression). **Table S5**: ROC curve analysis.

## Data Availability

We disclose any restrictions on the availability of data, materials and associated protocols. The datasets used and/or analysed during the current study are available from the corresponding author on reasonable request.

## Abbreviations

ACEF: Age, creatinine, ejection fraction score
ACS: Acute coronary syndrome
AUC: Area under the curve
CABG: Coronary artery bypass graft
GRACE: Global registry for acute coronary events score
MACE: Major adverse cardiac event
MI: Myocardial infarction
NSTE-ACS: Non-ST-elevation acute coronary syndrome
NSTEMI: Non-ST-segment elevation myocardial infarction
PCI: Percutaneous coronary intervention
ROC: Receiver operating characteristic
STEMI: ST-segment elevation myocardial infarction
SYNTAX: The Synergy Between Percutaneous Coronary Intervention with TAXUS and Cardiac Surgery score
UA: Unstable Angina
