# Supplementary figures and images for: ACEF performed better than other risk scores in non-ST-elevation acute coronary syndrome during long term follow-up
Source: BMC Cardiovasc Disord. 2021 Feb 3;21:70. doi: 10.1186/s12872-020-01841-2 (PMC7860189; doi:10.1186/s12872-020-01841-2)

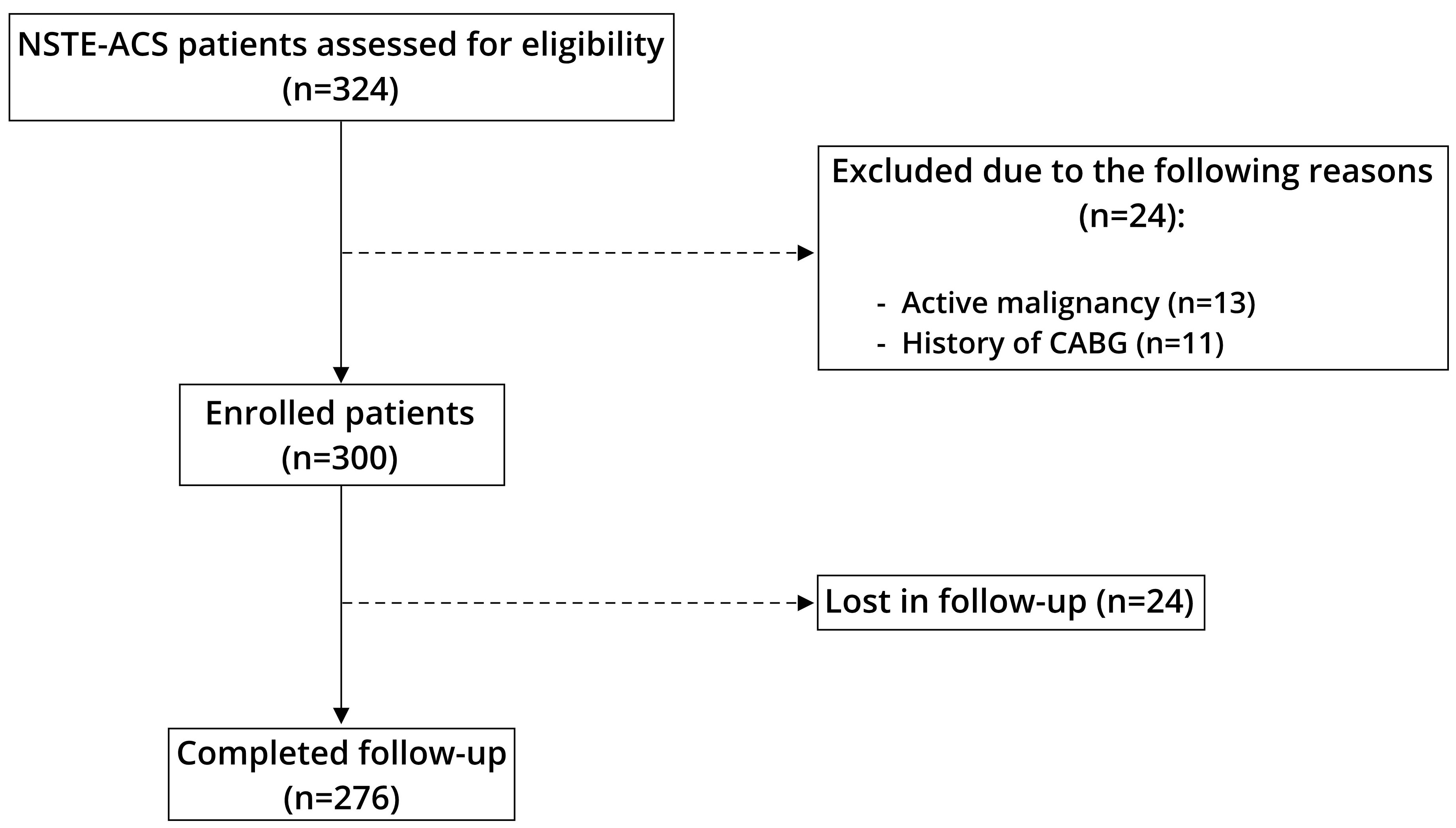

Supplement: Supplementary file 1 — Additional file 1. Fig. S1: Flow diagram of the study design. [file 12872_2020_1841_MOESM1_ESM.jpg]
