# Supplementary material for: ACEF performed better than other risk scores in non-ST-elevation acute coronary syndrome during long term follow-up
Source: BMC Cardiovasc Disord. 2021 Feb 3;21:70. doi: 10.1186/s12872-020-01841-2 (PMC7860189; doi:10.1186/s12872-020-01841-2)
Supplement: Supplementary file 2 — Additional file 2. Table S1: Overview of the first risk score validation in the setting of ACS or PCI. Table S2: Description of risk scores. Table S3: Association of risk scores with occurrence of major adverse cardiovascular events. Table S4: Comparison of predicted probabilities of ACEF and different computed models (derived from bivariate logistic regression). Table S5: ROC curve analysis. [file 12872_2020_1841_MOESM2_ESM.docx]

**Supplementary Material**

**Supplementary Figure 1. Flow diagram of the study design.**

**Legend:** NSTE-ACS – non-ST-elevation acute coronary syndrome; CABG – coronary artery bypass grafting.

**Supplementary Table 1** Overview of the first risk score validation in the setting of ACS or PCI

| **Risk score** | **Year** | **Population (number of patients)** | **Outcome** | **AUC** |
| --- | --- | --- | --- | --- |
| **GRACE [9]** | 2003 | ACS with and without ST-segment elevation (11389) | In-hospital mortality | 0.830 |
| **ACEF [21]** | 2011 | All-comers undergoing PCI (1208) | 1-year MACCE | 0.577 |
| **SYNTAX [14]** | 2009 | Patients with LM or 3-vessel CAD undergoing PCI or CABG (1800) | 1-year MACCE | / |
| **Clinical SYNTAX [15]** | 2010 | Patients with *de novo* multivessel CAD undergoing PCI (607) | 5-year MACCE | 0.690 |
| **SYNTAX II [16]** | 2013 | Patients with LM or 3-vessel CAD undergoing PCI or CABG (1800) | 4-year mortality | 0.725 |

Abbreviations: ACEF – age, creatinine and ejection fraction risk score; ACS – acute coronary syndrome; AUC – area under the ROC curve; CABG – coronary artery bypass graft; CAD – coronary artery disease; GRACE – Global Registry of Acute coronary events risk score; MACCE – Major adverse cardiac and cerebrovascular events; PCI – percutaneous coronary intervention; SYNTAX – The Synergy Between Percutaneous Coronary Intervention with TAXUS and Cardiac Surgery risk score.

**Supplementary Table 2** Description of risk scores

| **Risk score** | |
| --- | --- |
| **Variables** | **Points** |
| ***GRACE risk score*** | |
| Age (years) | <30 = 0 points; 30-39 = 8 points; 40-49 = 25 points; 50-59 = 41 points; 60-69 = 58 points; 70-79 = 75 points; 80-89 = 91 points; ≥90 = 100 points. |
| Heart rate (bpm) | <50 = 0 points; 50-69 = 3 points; 70-89 = 9 points; 90-109 = 15 points; 110-149 = 24 points; 150-199 = 38 points; >200 = 46 points. |
| Systolic blood pressure (mmHg) | <80 = 58 points; 80-99 = 53 points; 100-119 = 43 points; 120-139 = 34 points; 140-159 = 24 points; 160-199 = 10 points; >200 = 0 points. |
| Serum creatinine (µmol/L) | <35.3 = 1 point; 35.4-70 = 4 points; 71-105 = 7 points; 106-140 = 10 points; 141-176 = 13 points; 177-353 = 21 points; >354 = 28 points. |
| Cardiac arrest at admission | If yes = 39 points. |
| ST segment change on ECG | If yes = 28 points. |
| Abnormal cardiac enzymes | If yes = 14 points. |
| Killip class | Class 1 = 0 points; Class 2 = 20 points; Class 3 = 39 points; Class 4 = 59 points. |
| **Formula:** | **Sum of each individual points.** |
| ***ACEF risk score*** | |
| Age (years) | Points equal to numerical value.­­­­­­­ |
| LVEF (%) | Points equal to numerical value.­­­­­­­ |
| Serum creatinine (µmol/L) | If >176 = 1 point. |
| **Formula:** | $\frac{\boldsymbol{Age}}{\boldsymbol{LVEF}}\boldsymbol{+1 (If Creatinine>}\mathbf{176}\boldsymbol{\mu mol/L)}$ |
| ***SYNTAX risk score**** | |
| Affected coronary segment | Points weighted according to 16 segments and its variants. |
| Total occlusion | If yes = 5 points. |
| Characteristics of total occlusion | Blunt stump = 1 point; Bridging = 1 point; Sidebranch at the origin of the occlusion = 1 point; Some segments beyond occlusion visualized by contrast = 0-2 points; No segments beyond occlusion visualized by contrast = 3 points. |
| Trifurcation lesion | If yes: 1 diseased segment = 3 points; 2 diseased segments = 4 points; 3 diseased segments = 5 points; 4 diseased segment = 6 points. |
| Bifurcation lesion | If yes: Medina 1,0,0; Medina 1,1,0; Medina 0,1,0 = 1 point; Medina 1,0,1; Medina 1,1,1; Medina 0,0,1; Medina 0,1,1 = 2 points. |
| Aorto-ostial lesion | If yes = 1 point. |
| Severe tortuosity | If yes = 2 points. |
| Length >20 mm | If yes = 1 point. |
| Heavy calcification | If yes = 2 points. |
| Thrombus | If yes = 1 point. |
| Diffuse/small vessel disease | If yes = 1 point for each affected segment. |
| **Formula:** | **Risk score is calculated using an algorithm which accounts for affected coronary segments and other angiographic characteristics.** |
| ***Clinical SYNTAX risk score*** | |
| SYNTAX score (value) | Points equal to numerical value.­­­­­­­ |
| Age (years) | Points equal to numerical value.­­­­­­­ |
| LVEF (%) | Points equal to numerical value.­­­­­­­ |
| eGFR (mL/min) | For each 10 mL decrease of eGFR <60 mL/min/1.73 m^2^ = 1 point. |
| **Formula:** | $\boldsymbol{SYNTAX x}\left[ \frac{\boldsymbol{Age}}{\boldsymbol{LVEF}}\boldsymbol{+1 (for each 10 mL}\mathbf{decrease of eGFR}\boldsymbol{<60 mL/min/1.73 m}\boldsymbol{2} \right]$ |
| ***SYNTAX II PCI and SYNTAX II CABG*** | |
| SYNTAX score (value) | Risk scores are calculated using an algorithm which accounts for angiographic and clinical characteristics. |
| Age (years) |  |
| LVEF (%) |  |
| eGFR (mL/min) |  |
| Left main (yes/no) |  |
| Gender |  |
| COPD (yes/no) |  |
| PVD (yes/no) |  |

*Only lesions ≥50 % in a vessel ≥1.5 mm of diameter should be scored.

Abbreviations: ACEF – age, creatinine and ejection fraction risk score; CABG – coronary artery bypass graft; COPD – chronic obstructive pulmonary disease; eGFR – estimated glomerular filtration rate; GRACE – Global Registry of Acute coronary events risk score; LVEF – left ventricular ejection fraction; PCI – percutaneous coronary intervention; PVD – peripheral vascular disease; SYNTAX – The Synergy Between Percutaneous Coronary Intervention with TAXUS and Cardiac Surgery risk score.

**Supplementary Table 3** Association of risk scores with occurrence of major adverse cardiovascular events

| **Risk scores** | **MACE** | | **Total**  **(n=276)** | ***P***  **value*** |
| --- | --- | --- | --- | --- |
|  | **No**  **(n=212)** | **Yes**  **(n=64)** |  |  |
| **GRACE 2.0** |  |  |  | 0.109 |
| **<88.0** | 71 (33.5) | 16 (25.0) | 87 (31.5) |  |
| **89.0-118.0** | 92 (43.4) | 25(39.1) | 117 (42.4) |  |
| **>119.0** | 49 (23.1) | 23 (35.9) | 72 (26.1) |  |
| **ACEF** |  |  |  | 0.001 |
| **<1.0** | 74 (34.9) | 18 (28.1) | 92 (33.3) |  |
| **1.0-1.24** | 79 (37.3) | 13 (20.3) | 92 (33.3) |  |
| **>1.24** | 59 (27.8) | 33 (51.6) | 92 (33.3) |  |
| **SYNTAX** |  |  |  | 0.898 |
| **≤22** | 152 (71.7) | 44 (68.8) | 196 (80.1) |  |
| **23-32** | 47 (22.2) | 15 (23.4) | 62 (22.5) |  |
| **≥33** | 13 (6.1) | 5 (7.8) | 18 (6.5) |  |
| **Clinical SYNTAX** |  |  |  | 0.980 |
| **<10.42** | 71 (33.5) | 21 (32.8) | 92 (33.3) |  |
| **10.42-23.9** | 71 (33.5) | 21 (32.8) | 92 (33.3) |  |
| **>23.9** | 70 (33.0) | 22 (34.4) | 92 (33.3) |  |
| **SYNTAX II PCI** |  |  |  | 0.020 |
| **<22.7** | 79 (37.3) | 13 (20.3) | 92 (33.3) |  |
| **22.7-31.6** | 70 (33.0) | 22 (34.4) | 92 (33.3) |  |
| **>31.6** | 63 (29.7) | 29 (45.3) | 92 (33.3) |  |
| **SYNTAX II CABG** |  |  |  | 0.200 |
| **<20.6** | 75 (35.4) | 17 (26.6) | 92 (33.3) |  |
| **20.6-30.8** | 72 (34.0) | 20 (31.3) | 92 (33.3) |  |
| **>30.8** | 65 (30.7) | 27 (42.2) | 92 (33.3) |  |

Data are expressed as number (percentage).

*Chi-square test

**Abbreviations:** MACE – major adverse cardiovascular events; GRACE – Global Registry of Acute coronary events risk score; ACEF – Age, Creatinine and Ejection Fraction risk score; SYNTAX – The Synergy Between Percutaneous Coronary Intervention with TAXUS and Cardiac Surgery risk score; PCI – percutaneous coronary intervention; CABG – coronary artery bypass grafting

**Supplementary Table 4** Comparison of predicted probabilities of *ACEF* and different computed models (derived from bivariate logistic regression)

| **Variables** | **MACE** | | **Total**  **(n=276)** |
| --- | --- | --- | --- |
|  | **No**  **(n=212)** | **Yes**  **(n=64)** |  |
| **ACEF** | 0.207 (0.184-0.240) | 0.238 (0.194-0.266) | 0.212 (0.185-0.248) |
| **Model 1 (ACEF + FEM + AF)** | 0.173 (0.148-0.270) | 0.270 (0.179-0.357) | 0.189 (0.150-0.294) |
| **Model 2 (ACEF + FEM)** | 0.184 (0.158-0.269) | 0.244 (0.189-0.359) | 0.195 (0.160-0.290) |
| **Model 3 (ACEF + AF)** | 0.195 (0.174-0.230) | 0.227 (0.182-0.373) | 0.200 (0.176-0.241) |

Data are expressed as median predicted probabilities (interquartile range).

Abbreviations: ACEF – Age, Creatinine and Ejection Fraction risk score; FEM – female gender; AF – atrial fibrillation.

**Supplementary Table 5** ROC curve analysis

|  | **C-statistic (95% CI)** | **Sensitivity / specificity** | **p-value** |
| --- | --- | --- | --- |
| **ACEF** | 0.630 (0.548-0.713) | 60.9/67.9 | 0.002 |
| **Model 1 (ACEF + FEM + AF)** | 0.680 (0.602-0.758) | 71.9/61.3 | <0.001 |
| **Model 2 (ACEF + FEM)** | 0.666 (0.607-0.721) | 67.2/65.1 | <0.001 |
| **Model 3 (ACEF + AF)** | 0.631 (0.571-0.688) | 64.1/63.7 | 0.002 |

Data are expressed as median (interquartile range).

Abbreviations: ACEF – Age, Creatinine and Ejection Fraction risk score; FEM – female gender; AF – atrial fibrillation.
